# MXene-based solvent-responsive actuators with a polymer-intercalated gradient structure†

**DOI:** 10.1039/d4sc04935g

**Published:** 2024-12-03

**Authors:** Andi Di, Chenlu Wang, Yanlei Wang, Hongyan He, Wentao Deng, Pierre Stiernet, Lennart Bergström, Jiayin Yuan, Miao Zhang

**Affiliations:** a Department of Materials and Environmental Chemistry, Stockholm University Stockholm 114 18 Sweden lennart.bergstrom@mmk.su.se jiayin.yuan@mmk.su.se miao.zhang@mmk.su.se; b Beijing Key Laboratory of Ionic Liquids Clean Process, State Key Laboratory of Multiphase Complex Systems, CAS Key Laboratory of Green Process and Engineering, Institute of Process Engineering, Chinese Academy of Sciences Beijing 100190 China hyhe@ipe.ac.cn; c College of Chemistry and Chemical Engineering, Central South University Changsha 410083 China

## Abstract

Actuators based on electrically conductive and hydrophilic two-dimensional (2D) Ti_3_C_2_T_*X*_ MXene are of interest for fast and specific responses in demanding environments, such as chemical production. Herein, Ti_3_C_2_T_*X*_-based solvent-responsive bilayer actuators were developed, featuring a gradient polymer-intercalation structure in the active layer. These actuators were assembled using negatively charged pristine Ti_3_C_2_T_*X*_ nanosheets as the passive layer and positively charged polymer-tethered Ti_3_C_2_T_*X*_ as the active layer. 2D wide-angle X-ray scattering and simulations related the gradient polymer intercalated microstructure in the polymer/MXene composite active layer to the counterintuitive actuation behavior. The bending of the bilayer films in solvent vapor is triggered by the gradient polymer-intercalation and the differing diffusion rate of solvent molecules through the MX and MX-polymer layers of the bilayer actuator. With their ease of fabrication, remote light-control capabilities, and excellent actuation performance, the Ti_3_C_2_T_*X*_-based bilayer actuators reported here may find applications in areas such as sensors for monitoring chemical production, infrared camouflage, smart switches, and excavators in toxic solvent environments.

## Abstract 2

Solvent vapor-responsive Ti_3_C_2_T_*x*_-based actuators with a gradient microstructure in their composite active layer are prepared. The actuation behavior is understood by wide angle scattering, density functional theory and molecular dynamics.

## Graphical abstract

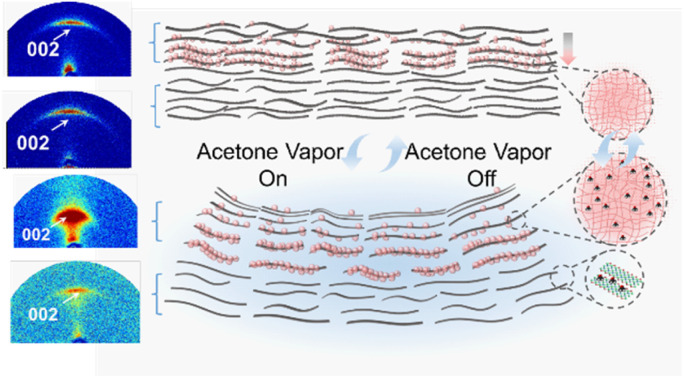

## Introduction

Lamellar membranes based on two-dimensional (2D) nanosheets with good flexibility, high electrical conductivity, and intercalated structures are of interest in a variety of applications, including flexible electrodes,^1^ separation membranes^2^ and adaptive devices.^3^ Electrically conductive actuators made from nanoscopic 2D building blocks assembled in tunable structures can convert external stimuli into motions, with potential uses in adaptive camouflage,^4^ haptics,^5^ medical devices,^6^ and sensors.^7^ The selection of materials, microstructural design, and the choice and optimization of fabrication methods are crucial factors in engineering actuators with advanced properties.

Various types of 2D nanomaterials, including chemically modified graphene,^8^ black phosphorus,^9^ molybdenum disulfide,^10^ and boron nitride,^11^ have been widely explored for integration into smart devices. MXenes, especially Ti_3_C_2_T_*X*_ (T_*X*_ represents various surface terminations, for example, –F, –OH, and/or =O), possess a unique combination of intrinsic properties, *e.g.* hydrophilicity and high electrical conductivity, making them highly suitable for the tunable assembly of nanostructured actuators.^12^ The hydrophilic surface of Ti_3_C_2_T_*X*_ facilitates the preparation of dispersions in aqueous and polar organic media and enables ionic complexation with macromolecules and surfactants, resulting in strong and flexible nanostructured materials.^13^ The high electrical conductivity of Ti_3_C_2_T_*X*_ eliminates the need for complicated post-reduction processes or the addition of conductive fillers, which are typically required for graphene oxide and other 2D materials.^14^ Consequently, pristine Ti_3_C_2_T_*X*_ and Ti_3_C_2_T_*X*_/polymer hybrid films have been actuated using humidity, near-infrared light, electricity, and heat.^4,15^ The photothermal properties of some MXenes have also been used for actuation,^4,16^ and the hydrophilic nature of MXene is crucial for the humidity response.^17^

Despite significant progress in enhancing the motion speed, stability, mechanical strength and multi-responsiveness of MXene-based materials, demonstrations of Ti_3_C_2_T_*X*_-based soft actuators remain sparse. In particular, Ti_3_C_2_T_*X*_-based actuating systems that respond to organic solvent vapours (OSVs), such as those used in chemical production,^18^ have been rarely investigated. Current OSV-responsive actuators, which include surface-patterned polymer films,^19^ porous polymer films,^20^ and bilayer polymer films,^21^ are limited by their poor electrical conductivity.

Previous studies on Ti_3_C_2_T_*X*_-based hybrid actuators have demonstrated that homogeneous films, produced *via* methods such as vacuum-assisted filtration can yield a relatively fast response to humidity.^15*a*^ Additionally, GO/Ti_3_C_2_T_*X*_ bilayer films produced by wet casting have been shown to respond to humidity, near-infrared light and electricity.^22^ It is evident that task-specific and rapid responses require improved structural control, as demonstrated by devices with gradient porosity^20*a*^ or compositional structures,^23^ which exhibit fast and specific actuation. To the best of our knowledge, while there are reports of Ti_3_C_2_T_*X*_-based gradient film actuators responding to light^16*b*^ and humidity,^24^ MXene-based gradient films designed for a fast response to organic solvent vapour have not been reported previously.

Here, we demonstrate Ti_3_C_2_T_*X*_ MXene-based bilayer actuators comprising a pristine MXene (MX) layer and a polymer-modified MXene (polymer-MX) composite layer. The bilayer film exhibited fast helical bending in organic solvent vapour, with the passive MX layer and the active polymer-MX layer. The actuation response of the MX/polymer-MX bilayer films contrasts with previously studied bilayers, where the active layer typically has higher swellability than the passive layer.^21*b*,25^ Small- and wide-angle X-ray scattering (SAXS and WAXS, respectively) indicated a gradient in the degree of polymer intercalation in the polymer-MX active layer, which may be responsible for the counterintuitive and fast bending behavior. Density functional theory (DFT) calculations and molecular dynamics (MD) simulations suggest that the differing diffusion rates of acetone vapor in the two layers of the actuators also contribute to the counterintuitive bending behavior of the film.

## Results and discussion

### Preparation and characterization of film actuators

**Fig. 1:**
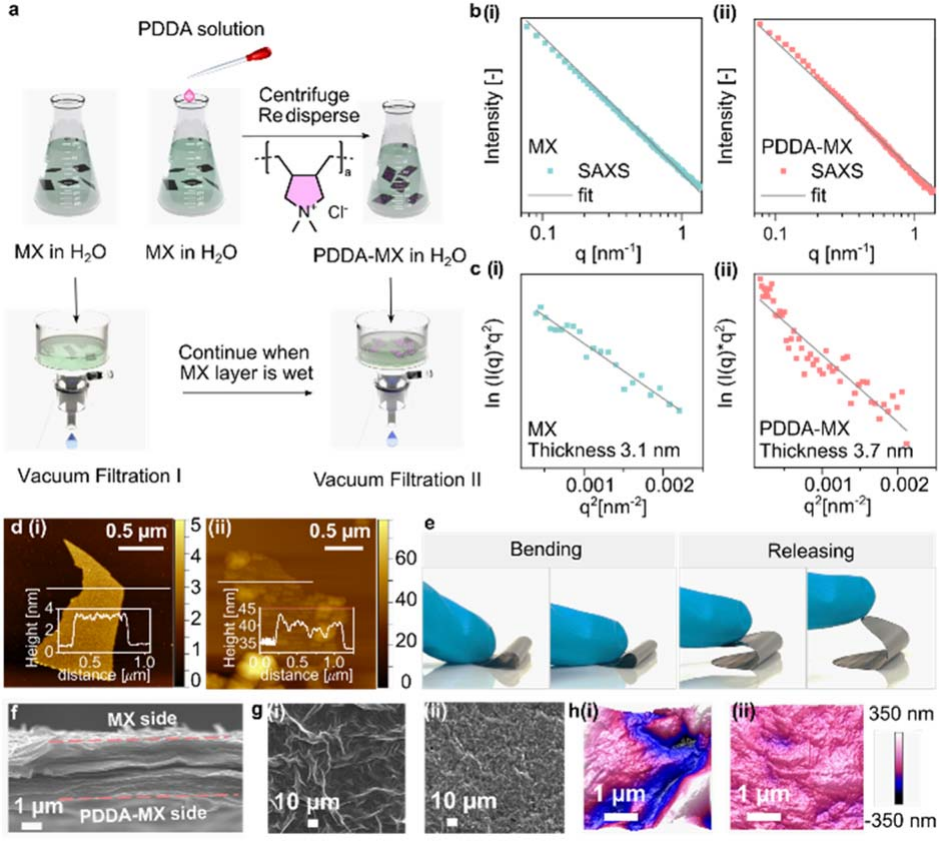
Design concept and structural characterization of the film actuators. (a) Schematic of the assembly process of the PDDA-MX/MX film actuator. (b) The 1D SAXS pattern of (i) the MX aqueous dispersion (2 mg mL^−1^) and (ii) the PDDA-MX aqueous dispersion (2 mg mL^−1^). (c(i) and c(ii)) Plot of In(*I*(*q*)*q*^2^) *versus q*^2^ of SAXS intensity in (b). (d) AFM images of (i) MX flakes and (ii) PDDA-MX flakes. (e) The bending and releasing of the PDDA-MX/MX film. (f) SEM image of the cross-section of a PDDA-MX/MX film. (g) SEM images of the surfaces of (i) a MX film and (ii) a PDDA-MX film. (h) 3D rendered AFM images of (i) a MX film and (ii) a PDDA-MX film.

The preparation of the poly(diallyldimethylammonium chloride)-modified MXene (PDDA-MX)/MX film actuator (Fig. 1a) begins with the preparation of the two individual aqueous dispersions of fully delaminated negatively charged Ti_3_C_2_T_*X*_ MXene (termed “MX”) and cationic poly(diallyldimethylammonium chloride)-modified Ti_3_C_2_T_*X*_ MXene (termed “PDDA-MX”). The delaminated MX is synthesised from Ti_3_AlC_2_ MAX powder by the well-established minimally intensive layer delamination method.^26^ The XRD pattern of MX powder (Fig. S1†) confirms the successful etching and delamination of the MAX phase. The Tyndall effect of the MX dispersion as shown in Fig. S2,† indicates its colloidal behaviour. The small angle X-ray scattering (SAXS) intensity of the MX aqueous dispersion (Fig. 1b(i)) gives a slope of *q*^−2^, characteristic of 2D materials.^27^ The absence of a correlation peak in the SAXS pattern (Fig. 1b(i)) indicates that MX flakes exist as single layers in the dispersion. The colloidal MX flakes have a hydrated thickness of 3.1 ± 0.7 nm according to the analysis of the SAXS signal (Fig. 1c(i)),^28^ which corresponds well to their thickness (∼2.5 nm) in a dry state obtained by atomic force microscopy (AFM) (Fig. 1d(i)). The transmission electron microscopy (TEM) image (Fig. S3†) further confirms that MX is delaminated.

The positively charged PDDA-MX was prepared by assembling a commercial polymer PDDA (average *M*_w_ = 400–500 kDa) onto the surface of MX *via* mixing in solution, as illustrated in Fig. 1a. A colloidal stable (Tyndall effect in Fig. S4 and the stability test in Fig. S5†) and monolayer-dispersed PDDA-MX dispersion was obtained, as confirmed by the power-law fitting of the SAXS signal (Fig. 1b(ii)) and using the TEM image (Fig. S3†). It indicates the molecular level complexation between the oppositely charged polymer and MX flakes. In the colloidal state, the PDDA-MX flakes in a hydrated form have an average thickness of 3.7 ± 0.9 nm (Fig. 1c(ii)). The AFM image (Fig. 1d(ii)) of PDDA-MX flakes in a dry state shows a nanoscale patchy surface with uneven PDDA aggregates anchoring onto the MX surface, showing a thickness range between 2.5 nm and 6.0 nm. This co-assembly of PDDA with MX flakes is ascribed to the electrostatic interactions and the random distribution of polar surface terminations of MX flakes. The presence of PDDA is further supported using the Fourier transform infrared spectrum (FTIR, Fig. S6†).

Assembly of the bilayer actuator includes a sequential two-step filtration process on a PVDF membrane substrate (pore size, 0.25 μm), as shown in Fig. 1a. A pristine MX aqueous dispersion is filtered until a gel state is reached, followed by the addition of a PDDA-MX dispersion to obtain a free-standing bilayer film, abbreviated as PDDA-MX/MX, which shows adequate mechanical flexibility (Fig. 1e). The cross-section of the PDDA-MX/MX film shows a well-aligned lamellar morphology with a thickness of *ca.* 2.8 μm (Fig. 1f). Both SEM (Fig. 1g) and AFM images (Fig. 1h) suggest that the pristine MX film has more blobs (height of *ca.* 600 nm, Fig. S7†) at the surface than the PDDA-MX film.

### Performance of the bilayer actuator and multi-responsiveness

**Fig. 2:**
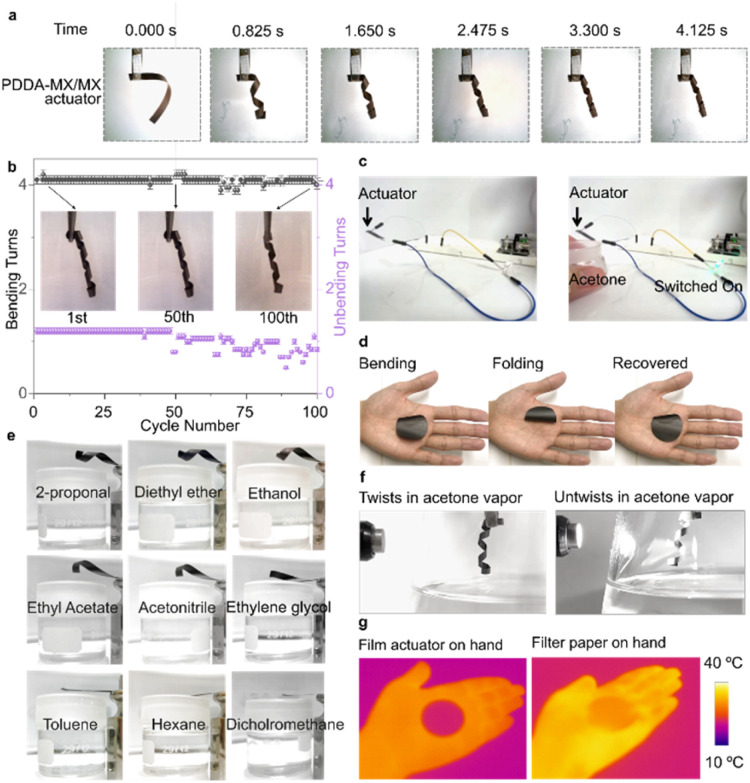
Multi-responsiveness of the PDDA-MX/MX bilayer actuators. (a) Time course profiles of the helical bending of the PDDA-MX/MX film. (b) Reversible bending and unbending of the PDDA-MX/MX film in acetone vapor for 100 cycles. (c) PDDA-MX/MX film actuator used as a controllable switch in a circuit to indicate acetone vapor stimulation. (d) Oscillation of the PDDA-MX/MX film actuator on a hand. (e) Motions of the PDDA-MX/MX film actuator to different organic vapors. (f) The film actuator unbends when light is applied. (g) IR images showing the IR camouflage capability of the PDDA-MX/MX film.

The PDDA-MX/MX film was trimmed into rectangular strips *ca.* 45° relative to the anisotropic axis of the film which was indicated by the line pattern of the used PVDF membrane (Fig. S8a†). These strips were used for testing. When moved from the ambient air into an acetone vapor environment, the PDDA-MX/MX strip exhibits a rapid bending from the MX-based passive layer to the PDDA-MX active layer (two circles within 1 s, Movie S1†). The actuation of the bilayer that was prepared on a thin CNF membrane (PDDA-MX/MX_(CNF)_) displayed concentric bending (Fig S8b†), which indicates that the helical nature of bending of the bilayers prepared on a thin PVDF membrane may be related to an anisotropic structure of the PVDF filtration membrane (Fig. S8a†). The time-dependent bending procedure is illustrated in Fig. 2a. Fast recovery of bending was also observed in Movie S1.† The durability test for 100 bending/unbending cycles of the PDDA-MX/MX film (Fig. 2b) shows no decrease in the bending loops for the first 50 cycles, and a small decrease for the 100th cycle, as illustrated in the inset. There are some fluctuations in the unbending loops in the last 40 cycles, which may be caused by the subtle microstructural changes induced by the multiple intercalation and deintercalation during cycling. The actuation performance in 97% relative humidity (RH) is much slower than that in the acetone vapour, as illustrated in Fig. S9.†

Compared to other materials, such as graphene oxide, cellulose and polyvinyl alcohol,^22,29^ a primary advantage of MXene-based actuators is their intrinsic high electrical conductivity. Therefore, we tested its function as a vapour-controllable electrical switch. As shown in Fig. 2c, when a container with acetone approaches, the PDDA-MX/MX film bends to connect the circuit and lights up the LED lamp (Movie S2†). Once acetone vapour is removed, the PDDA-MX/MX film unbends to switch off the lamp.

Furthermore, the PDDA-MX/MX film actuator delivers a fast response to moisture, as evidenced by its oscillating movements when placed on a human hand (Movie S3†). When placed on the hand, the film bends and folds from the surface, and recovers once it is away from the hand surface (Fig. 2d). Next, the actuation tests are extended to other solvents. The film bends helically when placed over the air-solvent interfaces, as illustrated in Fig. 2e. Only organic solvents with low polarity (refer to Table S1†), *e.g.* toluene and hexane, produce expectedly no actuation. Additionally, the bending performance of the PDDA-MX/MX film actuator in the vapors of diethyl ether, ethyl acetate, acetonitrile, and ethanol is compared in Fig. S10.† Among these organic solvents, the film actuator exhibits the fastest bending response in diethyl ether, likely due to a combination of the solvent's polarity and saturated vapour pressure.

Apart from organic vapours, the PDDA-MX/MX film actuator also bends in liquid acetone (Movie S4†). This behaviour demonstrates the robustness of the assembled layered structure due to the insolubility of PDDA (Fig. S11†) and MXene in acetone, and strong adhesion between oppositely charged Ti_3_C_2_T_*X*_ and PDDA. Beyond responding to organic solvents and humidity, the actuation is also light-controllable. The film actuator unbends to some extent when point light is applied, warming the film and desorbing solvent molecules from the film, as recorded in Movie S5† and illustrated in Fig. 2f. The light-responsive capability enables this actuator to function as a remotely controllable smart device. Additionally, the film exhibits IR camouflage properties, as shown in Fig. 2g.

### Experimental and computational investigations of the unusual actuation mechanism

**Fig. 3:**
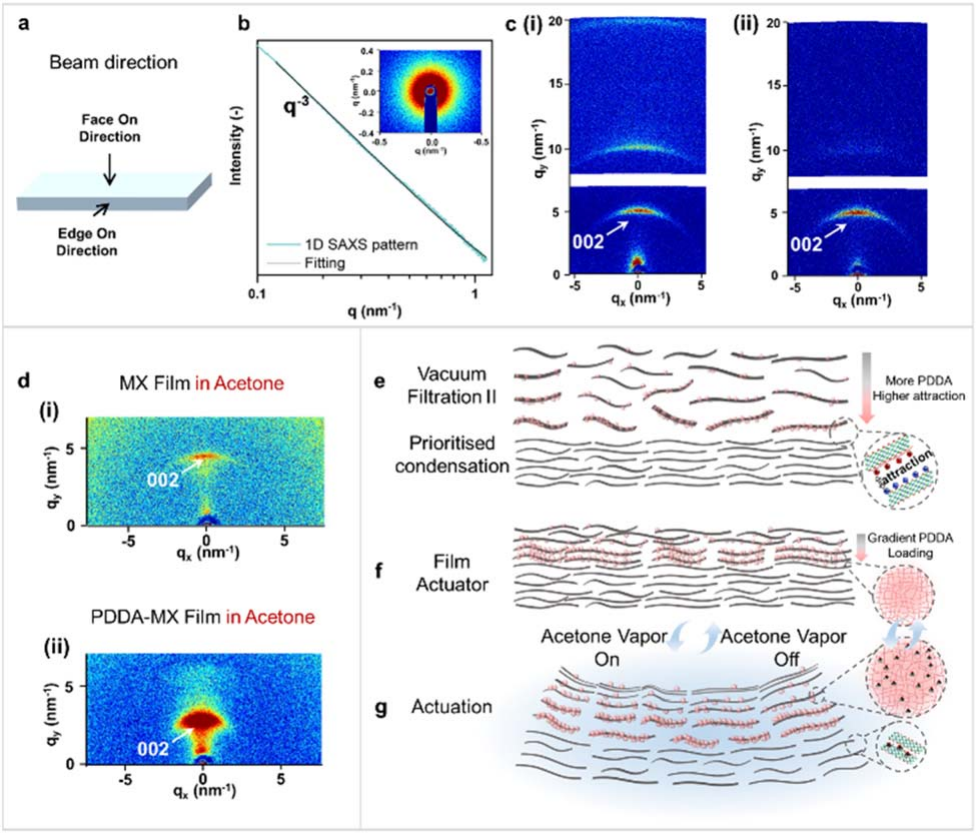
SAXS and WAXS characterization and the actuation mechanism. (a) Illustration of the two directions in which the X-ray beam was applied to the film. (b) Representative 1D face-on SAXS pattern of the MX film, the inset shows the 2D SAXS pattern. (c) 2D edge-on WAXS pattern of (i) the dry MX film and (ii) the dry PDDA-MX film. (d) 2D edge-on WAXS patterns of (i) the MX film and (ii) the PDDA-MX film in liquid acetone. (e–g) Actuation mechanism shown from the side view of the PDDA-MX/MX film actuator. The grey rectangles represent the MX flakes. The pink spheres represent PDDA macromolecules. (e) The preferable stacking of more positively charged PDDA-MX flakes onto the negatively charged MX layer during vacuum-assisted filtration II in Fig. 1a. (f) The structure of the prepared film actuator with a gradient PDDA loading in the active layer. (g) Schematic deformation mechanism of the PDDA-MX/MX film actuator when exposed to acetone vapor.

The actuation performance of the pristine MX film and the PDDA-MX film was tested. Time course profiles of their actuation processes are listed in Fig. S12.† These results indicate that the fast actuation of the PDDA-MX/MX film in acetone vapour is attributed to the multi-scale structural heterogeneity of the as-assembled film, more complex than the common bilayers. SAXS and WAXS tests were conducted to investigate the structures of the individual MX film and PDDA-MX film, as well as their structural changes when in contact with liquid acetone, from both face-on and edge-on directions (Fig. 3a). The isotropic 1D SAXS pattern (Fig. 3b), in the face-on direction, could be fitted to a power-law exponent of *q*^−3^, which is typically observed for self-affine surfaces.^30^ The interlayer distance and the orientation degree of the MX flakes were measured in the edge-on direction using WAXS (Fig. 3a); the set-up of the measurement is illustrated in Fig. S13.† The edge-on WAXS patterns of both the dry MX film (Fig. 3c(i)) and the dry PDDA-MX film (Fig. 3c(ii)) show multiple anisotropic scattered rings, reflecting a preferred orientation of MX flakes, as expected. The sharp and narrow (002) scattered rings of both dry films suggest a low polydispersity in the interlayer distance (∼1.23 nm). On the basis of the fitting of the azimuthal angle plot (Fig. S14†), the orientation degree (π) of the MX flakes in the MX film and PDDA-MX film is calculated to be *ca.* 0.83 for both, using equation π = (180 − FWHM)/180, where FWHM is the full width at half maximum of the azimuthal angle curve. It indicates that the modification using the PDDA polymer does not affect the MX orientation in the film.

Fig. 3d shows the scattering patterns from the edge-on direction of the MX film and PDDA-MX film in liquid acetone. The organisation of both the MX film and PDDA-MX film is susceptible to acetone, as compared to Fig. 3c. The WAXS pattern of the MX film in acetone has a narrow anisotropic scattering ring (Fig. 3d(i)), with the (002) intensity range between *q*_*y*_ = 4.29 and 4.76 nm^−1^ and the highest intensity located at *q*_*y*_ = 4.52 nm^−1^, corresponding to an average interlayer distance of 1.39 nm and thus an acetone-responsive swelling degree of 12.1% compared to that of the dry MX film (1.24 nm, Fig. 3c(i)). This narrow ring in the 2D WAXS pattern for the MX film (Fig. 3d) shows a relatively homogeneous swelling of interlayer distance when exposed to acetone.

In contrast, as seen in Fig. 3d(ii), the WAXS pattern of the PDDA-MX film in acetone shows a broad anisotropic (002) ring with continuous intensity in the *q*_*y*_ range between 1.88 and 5.87 nm^−1^. This corresponds to a stacking distance of MX flakes ranging from 3.34 to 1.07 nm, as a consequence of inhomogeneous swelling of different parts of the PDDA-MX composite active layer in liquid acetone. The relatively higher scattering intensity in the low *q*_*y*_ range indicates a greater proportion of larger interlayer distances. The largest interlayer distance of the acetone-swelled PDDA-MX film is approximately 2.4 times the average distance of the acetone-swelled MX film (∼1.39 nm). Based on WAXS characterization results, the PDDA-MX layer exhibits a gradient PDDA-intercalated microstructure responsible for the broad anisotropic (002) ring in the 2D WAXS pattern. This microstructural gradient results in inhomogeneous swelling, which in turn causes the counterintuitive bending from the MX side (passive layer) to the PDDA-MX composite side (active layer).

As illustrated in Fig. 3e and f, the formation of this gradient PDDA-intercalated microstructure is attributed to two main factors: (1) the wide distribution in the degree of PDDA coverage on MX nanosheets in the precursor PDDA-MX sample, as visualised in the AFM images of the PDDA-MX precursor in Fig. S15,† which results in variations in charge density of PDDA-MX flakes; (2) the PDDA rich-MX flakes, which have more positive charges, preferentially stack on top of the negatively charged MX gel layer during the vacuum-assisted filtration step (Fig. 1a), driven by the electrostatic interactions. This is supported by their zeta potentials (Fig. S16†) and the immediate agglomeration observed when MX dispersion and PDDA-MX dispersion are mixed (shown in Movie S6†). Thus, a gradient PDDA-intercalated microstructure is formed with a decreasing amount of PDDA from the interface of the two layers to the outer surface of the PDDA-MX layer (the air/PDDA-MX interface). When adsorbing acetone vapor, the interlayer expansion of the interface between the MX and PDDA-MX layers is the largest compared to regions further from the interface, as illustrated in Fig. 3g. This causes rapid bending with the swelling PDDA-MX layer bending inwards, resulting in swelling-induced opposite bending.

**Fig. 4:**
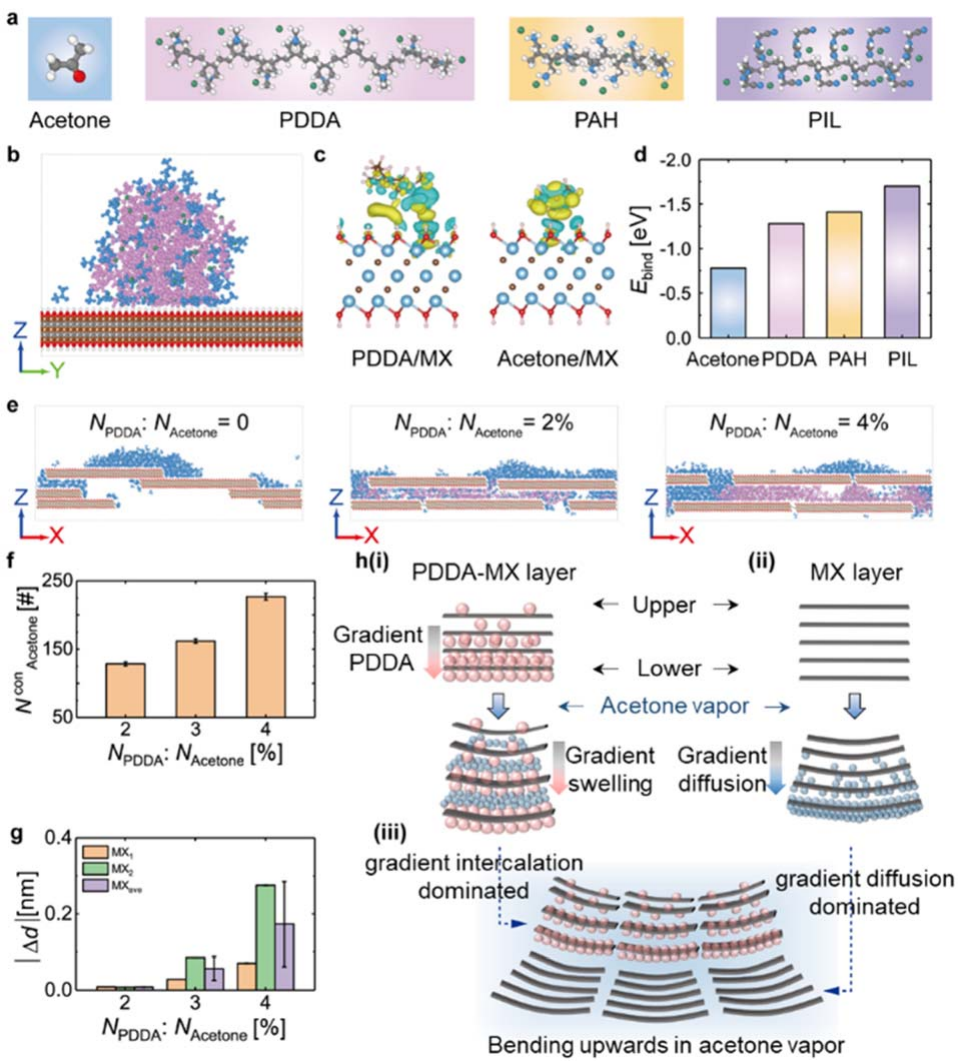
Theoretical simulations of polymer-MX-acetone composite systems. (a) Atomic structures of acetone and three polycations of PDDA, PAH, and PIL, where white, grey, red, and blue colors represent H, C, O, and N atoms, respectively, and green color represents Cl^−^ or Br^−^. (b) The equilibrium configuration of the acetone-doped PDDA droplet on the MX surface, where pink, green, and blue colors represent the polycation of PDDA, the counteranion of PDDA, and acetone, respectively. (c) The CDD of PDDA/MX and acetone/MX, where yellow and blue colors represent the accumulation and depletion of charge, respectively. The iso-surface level is 0.0007 e Å^−3^. (d) The binding energy (*E*_bind_) of acetone/MX, PDDA/MX, PAH/MX, and PIL/MX. (e) The equilibrium configuration of acetone molecules inserted into the nanochannel of MX or PDDA-MX. (f) The number of acetone molecules inserted into the PDDA-MX (*N*_Acetone_^con^). (g) The change of |Δ*d*| *versus* the *N*_PDDA_ : *N*_Acetone_ ratio. MX_1_ and MX_2_ represent the |Δ*d*| values at two positions (as marked in Fig. S18†), and MX_ave_ represents the average values. (h) Actuation mechanisms of the PDDA-MX/MX film actuator when exposed to acetone vapor, where the grey rectangles represent the cross-section of the MX flakes, and the pink spheres represent the PDDA molecules. (i) Due to the gradient distribution of PDDA molecules, acetone molecules (blue spheres) are inserted into the interlayer quickly, causing the PDDA-MX layer to bend upwards. (ii) Acetone molecules (blue spheres) are preferentially adsorbed on the MX surface, causing the MX layer to bend upward. (iii) Combining (i) and (ii), the film actuator bends with the PDDA-MX layer inwards when exposed to acetone vapor (blue shadow).

MD simulations and DFT calculations were performed to further understand the actuation mechanism of the PDDA-MX/MX film in acetone vapour. The atomic structures of different components and their assigned colour are presented in Fig. 4a. First, the interactions between PDDA, acetone and MX are studied. Fig. S17a† illustrates the equilibrium configuration of a pure PDDA droplet-wetted MX surface, where large voids are formed in the PDDA due to the steric hindrance of polycations. After the uptake of acetone (Fig. 4b & S17b†), the PDDA droplet effectively adsorbs acetone molecules by filling the voids. Note that acetone tends to be adsorbed near PDDA rather than the MX surface. The charge density difference (CDD) (Fig. 4c & S18†) and the binding energy (Fig. 4d) results show that the charge transfer and structural stability of PDDA/MX are both stronger than those of acetone/MX, thus PDDA can stably anchor on the MX surface even in the presence of acetone.

The internal dominating actuation mechanism was then understood by insertion behaviors of acetone molecules into the MX nanochannels and the PDDA-MX nanochannels. Fig. S19a† shows acetone molecules (*N*_Acetone_ = 500) initially positioned above the pre-equilibrium PDDA-MX filled with different PDDA chains (*N*_PDDA_ = 0, 10, 15, and 20, corresponding to a *N*_PDDA_ : *N*_Acetone_ ratio of 0, 2%, 3% and 4%, respectively). As displayed in Fig. 4e & S19b,† acetone molecules prefer to adsorb on the MX surfaces. The presence of PDDA molecules in the PDDA-MX layer facilitates the rapid insertion of acetone molecules into PDDA-MX nanochannels instantly. Additionally, a higher concentration of *N*_PDDA_ leads to the insertion of more acetone molecules into PDDA-MX (parameterised using *N*^con^_Acetone_) (Fig. 4f), resulting in a greater expansion in the interlayer spacing. The absolute changes of the interlayer spacing of PDDA-MX (or MX) following acetone insertion (Δ*d*) are summarised in Fig. 4g. These simulated results offer a reasonable explanation for the experimental observation.

Based on the computer simulations, the actuation process of PDDA-MX/MX, as concluded from the experimental results, is developed and illustrated in Fig. 4h. The actuation of the PDDA-MX layer is primarily driven by gradient intercalation (Fig. 4h(i)). The presence of PDDA intercalated into the MX channels significantly facilitates and accelerates the insertion of acetone molecules. The upper side, with less *N*_PDDA_, adsorbs fewer acetone molecules (*N*^con^_Acetone_), resulting in a smaller |Δ*d*|. In contrast, the |Δ*d*| on the lower side of the PDDA-MX layer changes significantly, causing the lower side to bend towards the upper side of the layer. However the bending of the MX layer is dominated by the gradient acetone diffusion from the lower side to the upper side (Fig. 4h(ii)), as acetone is more easily adsorbed and accumulated on the MX outer surface (Fig. 4h(ii)). Moreover, the higher orientation of MX combined with compact lamellar packing, which lacks a polymer spacer, results in slower acetone diffusion (Movie S7, ESI†). As a result, the lower side of the MX layer adsorbs more acetone molecules than the upper side, forcing the lower side to bend towards the upper side. This behaviour is similar to that observed in a homogeneous MXene film actuator exposed to humidity on one side.^15*a*^ Collectively, both the PDDA-MX layer and the MX layer exhibit upward bending behaviour, though the underlying mechanisms differ. The combined effects of these distinct mechanisms lead to the counterintuitive actuation behavior observed in the PDDA-MX/MX film actuator. This explains why, upon contact with solvent molecules, the PDDA-MX layer, which has a higher degree of swelling, bends inwards. This behaviour is contrary to the typical behaviour of common bilayers, where the passive layer with a lower degree of swelling usually bends inwards.

### Tunability of actuation performance with diverse polycations

In addition to the PDDA polycation, this method is robust and applicable to other common polycations. This general concept is demonstrated here by using commercial poly(allylamine hydrochloride) (PAH) and self-synthesised poly(ionic liquid) poly(1-cyanomethyl-3-vinylimidazolium bromide) (PIL, ^1^H-NMR in Fig. S20†) to prepare PAH-MX/MX and PIL-MX/MX film actuators. Both FTIR (Fig. S21†) and elemental mapping (Fig. S22 and S23†) confirm the successful assembly of PAH and PIL with MX in their respective dispersions. Similarly, PAH and PIL appear as small particles on the surface of MX flakes, as shown in their AFM images (Fig. S24†). The PIL-MX and PAH-MX films exhibit flatter surfaces compared to the MX film, similar to the PDDA-MX film (Fig. S25†).

**Fig. 5:**
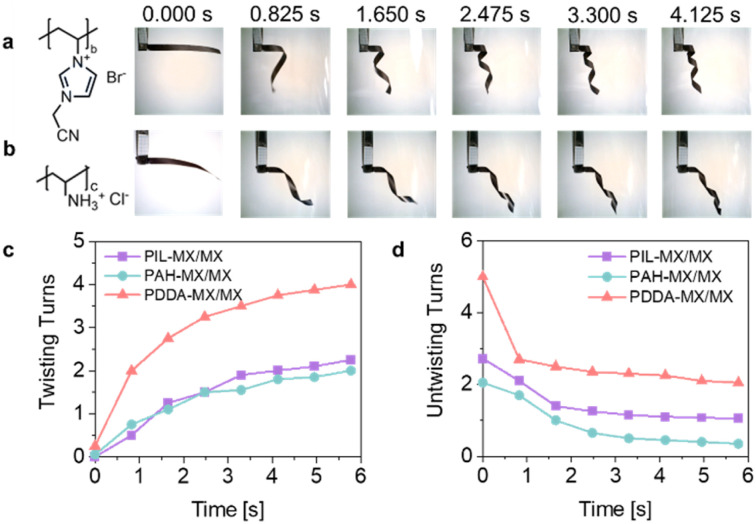
Programmable actuation speed. Time course profiles of the helical bending movements of (a) the PIL-MX/MX bilayer actuator and (b) the PAH-MX/MX bilayer actuator. Comparison of time-dependent (c) bending actuation triggered by acetone vapour and (d) unbending actuation after the removal of acetone vapour.

The first 5.7 s of the time-dependent helical bending of PIL-MX/MX and PAH-MX/MX film actuators are shown in Fig. 5a and b (Movie S8 and S9†). As compared in Fig. 5c, the PDDA-MX/MX actuator exhibits the fastest actuation speed in acetone vapour. This variation is attributed to differences in the polymer loading amounts and their swelling capabilities with acetone molecules. According to the TGA results in Fig. S26,† the average loading amount of PDDA onto MX flakes is 12.3 wt%, which is higher than the loading amounts of PAH (4.8 wt%) and PIL (6.2 wt%). This difference in loading is presumably related to the variations in the charge density and rigidity of the polymer backbones. The unbending speed is also compared in Fig. 5d when the strip actuator is removed from the acetone vapor into air. Fast recovery occurs during the first second, and the PDDA-MX/MX film actuator shows the fastest recovery speed, followed by PIL-MX/MX and PAH-MX/MX film actuators.

The wetting behaviour of the three polymer droplets was simulated for comparison. Fig. S27a† illustrates the morphology of the PAH and PIL droplets on the MX surface, which is similar to that of the PDDA droplet. In acetone vapour, acetone molecules were adsorbed around the three polymer droplets, leading to the rearrangement of polycations and counteranions (Fig. S27b†). Furthermore, we found that the *E*_bind_ between the three polymers and the MX surface is stronger than that between acetone and the MX surface. As illustrated in Fig. 4d, the binding strength follows the order: acetone/MX < PDDA/MX < PAH/MX < PIL/MX. This indicates that the three polymers have a greater affinity for adsorbing onto the MX surface compared to acetone. In general, a more negative value in *E*_bind_ between polymers and the MX surface indicates greater stability of the polymer/MX structure. Consequently, acetone exerts less influence on the structure, leading to a slower helical bending speed. This trend aligns with the experimental observations shown in Fig. 5c.

**Fig. 6:**
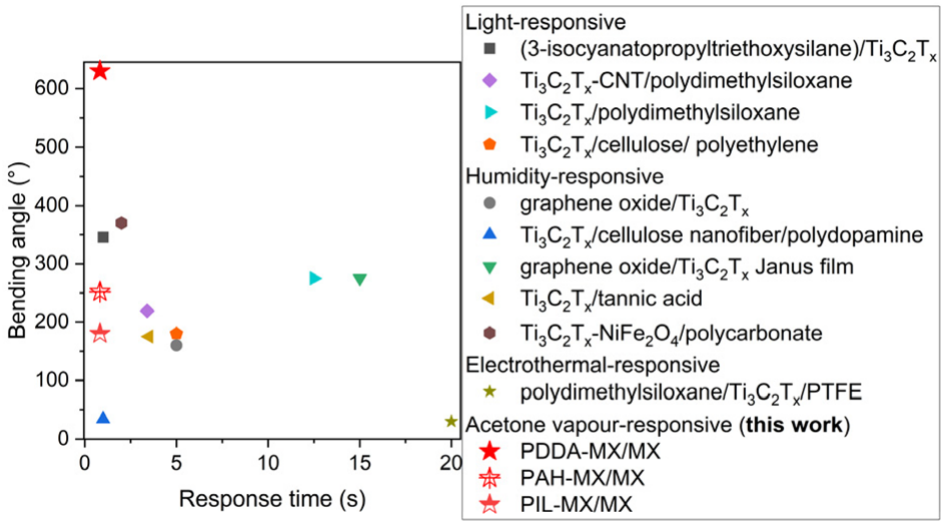
Comparison of actuating performances of Ti_3_C_2_T_*X*_-based actuators that respond to different stimuli.

Overall, the Ti_3_C_2_T_*X*_-based bilayer films, with one layer featuring gradient polymer intercalation, exhibit fast actuation performance upon contact with acetone vapour. These films either outperform or are on par with other light/humidity/electrothermal responsive Ti_3_C_2_T_*X*_-based actuators reported previously,^22,24,31^ as compared in Fig. 6. These actuators have potential applications for smart devices, *e.g.* smart windows for cooling and evacuation.

## Experimental

### Materials

Poly(diallyldimethylammonium) chloride solution (PDDA, average *M*_w_ = 400 000–500 000, 20 wt% in water) was purchased from Sigma-Aldrich and was used without further purification. Poly(allylamine hydrochloride) (PAH, average *M*_w_ = 60 000) was purchased from Polysciences. Poly(1-cyanomethyl-3-vinylimidazolium bromide) (PIL, average *M*_w_ = 118 500) was synthesized according to a reported method.^32^ Hydrophilic polyvinylidene fluoride membranes (PVDF membrane, pore size 0.22 μm) were purchased from Sigma-Aldrich.

### Determination of molecular weight of the synthesized PIL

Molar masses (*M*_n_ and *M*_w_) and dispersity (*Đ*) of poly(1-cyanomethyl-3-vinylimidazolium bromide) (PIL) were characterized by size exclusion chromatography (SEC) at 25 °C in aqueous sodium acetate (0.1 M)/acetic acid (0.1 M) buffer (pH = 4.5) (DMF) containing MeOH (20 vol%) at a flow rate of 1 mL min^−1^ with a poly(2-vinylpyridine) calibration. SEC curves were recorded with an Agilent 1260 Infinity II equipped with two columns (PSS NOVEMA Max analytical Linear S and XL), a variable wavelength detector and refractive index detectors.

### Preparation of water-stable Ti_3_C_2_T_*X*_ colloidal dispersion

Ti_3_C_2_T_*X*_ was synthesized by etching MAX (Ti_3_AlC_2_) powder with a mixture of LiF and HCl solution. 2.5 g of LiF was dissolved in 30 mL of 9 M HCl in water, followed by the slow addition of 1.5 g of Ti_3_AlC_2_ (400 mesh) powder. The mixture was stirred at 500 rpm for 24 h at 35 °C. Upon completion of the reaction, the mixture was diluted with 200 mL of deionized water and centrifuged at 12 000 rpm for 1 h. The upper supernatant was decanted to retain the sediment. This washing process was repeated 4 times to obtain a slurry sediment. The slurry sediment was then dispersed in deionized water by shaking using a Vortex-Genie 2 mixer for 20 min, followed by centrifugation at 12 000 rpm for 1 h. The supernatant was discarded. This process was repeated 5 times to obtain successfully exfoliated Ti_3_C_2_T_*X*_. The as-exfoliated Ti_3_C_2_T_*X*_ in water was sonicated under a nitrogen-protective atmosphere for 10 min and then centrifuged at 3000 rpm for 20 min to remove the unexfoliated Ti_3_C_2_T_*X*_ particles. The yield of the Ti_3_C_2_T_*X*_ at 35 °C was 30.4 ± 4.0%.

### Preparation of polymer-modified MXene flake colloidal dispersion

PDDA-MX and PAH-MX were prepared by adding 10 grams of 2 wt% of polymer solution (PDDA or PAH) dropwise into 80 mL of MXene colloidal dispersion (0.5 mg mL^−1^), followed by stirring for 5 min. After the complexation process, the mixture was subjected to centrifugation at a speed of 12 000 rpm twice to eliminate any unbound polymers. Regarding the preparation of PIL-MX, 75 milligrams of 5 mg mL^−1^ MX dispersion was added to 40 mL of a 3.5 mg mL^−1^ PIL solution dropwise, stirred for 5 min and then centrifuged twice at 12 000 rpm to remove the unbound polymer in the system to prepare PIL-MX.

### Preparation of film actuators

To prepare the Ti_3_C_2_T_*X*_ (MX) film, 10 mL of a 1 mg mL^−1^ Ti_3_C_2_T_*X*_ dispersion was vacuum filtered onto a PVDF membrane to form the first layer. The second layer was created by applying 1 mL of a 5 mg mL^−1^ polymer-modified Ti_3_C_2_T_*X*_ dispersion on top of the Ti_3_C_2_T_*X*_ layer. The resulting film was then peeled off from the PVDF membrane for further characterisation. The actuation speed was tested using rectangular strips measuring 3 mm × 30 mm, cut from the prepared films, and evaluated at 20 °C in acetone vapor (saturated vapour pressure, 24.478 kPa). The strips were cut at approximately 45° relative to the anisotropic axis of the film, as indicated by the line pattern on the PVDF membrane.

### Preparation of films for small/wide angle X-ray scattering

A Ti_3_C_2_T_*X*_ MXene (MX) film and a PDDA-Ti_3_C_2_T_*X*_ (PDDA-MX) film were prepared separately by vacuum filtration to further investigate their structures using small/wide angle X-ray scattering.

### Characterisation

Small/wide angle X-ray scattering (SAXS/WAXS) was used to characterize MX dispersion, PDDA-MX dispersion, the MXene film and the PDDA-MX film on an Anton-Paar SAXSpoint 2.0 instrument equipped with a copper source (Cu Kα *λ* = 1.542 Å) and a 2D EIGER R series Hybrid Photon Counting (HPC) detector. The distance between the sample holder and detector for SAXS and WAXS measurements was 556 mm and 111 mm, respectively. The data were collected at 25 °C in one frame, with 300 s exposure time. The dispersion samples were prepared by loading a dispersion (2 mg mL^−1^) into a glass capillary with a diameter of 1.5 mm, and were then mounted on a multi-capillary sample holder. The solid film samples prepared by filtration were cut into a 1.5 mm strip and mounted on a solid sample holder for the structural studies in the dry state from both face-on and edge-on directions in transmission mode. For the investigation of structural changes when the films are exposed to acetone, the film strips were mounted in glass capillaries that were filled with acetone solvent. The sample-loaded capillaries were sealed using wax and mounted on a capillary sample holder for SAXS and WAXS measurements from both face-on and edge-on directions.

The phase of the MAX powder and the exfoliated MXene single layer was characterized using powder X-ray powder diffraction (PXRD, PANalytical) in the 2*θ* range between 3° and 70°. Atomic force microscopy (AFM, dimension 3100, Bruker, USA) was performed in PeakForce tapping mode. The scan size and rate were 2 × 2 μm^2^ and 2.0 Hz, respectively. The zeta-potential of the Ti_3_C_2_T_*X*_ (MX) flakes and polymer-modified Ti_3_C_2_T_*X*_ (PDDA-MX) flakes was studied using a Malvern Zetasizer Nano ZSP (Malvern, UK). The polymer-modified MXenes were characterized by Fourier-transform infrared spectroscopy (a Varian 610-IR FT-IR spectrometer) and Raman spectroscopy (LabRAM HR 800). Thermal gravimetric analysis was performed on a discovery TG analyzer (TA Instrument, U.S.) with a ramp rate of 5 °C min^−1^ from 20 to 600 °C underan Ar atmosphere (gas flow speed 30 mL min^−1^). Fourier transform infrared spectra (FT-IR) were obtained on an FT-IR spectrometer, Varian 610. Scanning electron microscopy (JEOL JSM-7000F) was used to observe the morphologies of the prepared films. The video of the actuation process was recorded using a microscope (Dino-Lite). Videos that capture the actuation process of the strips were converted to image sequences using VirtualDub software. Transmission electron microscopy (Jeol JEM 2100) was used to characterize the MX flakes and PDDA-MX flakes.

### Molecular dynamics (MD) simulations

As shown in Fig. S13a,† the liquid/solid interface built for the wetting system was constructed by placing a PDDA droplet near the MXene (MX) surface, where the PDDA droplet contains 6 unit chains, the atomic structures of PDDA are shown in Fig. 4a. The in-plane (*x*–*y*) size of the MX surface was set as 6.45 × 6.15 nm^2^, and the size of the initial three polymer droplets was 4.0 × 4.0 × 4.0 nm^3^. To reveal the influence of doping acetone on the composite system, the polymer droplet doped with 60 acetone molecules was established for comparison (Fig. S13b†).

Different from the wetting system, the initial configuration of the two-dimensional (2D) confined system is to put different chains of PDDA (*N*_PDDA_ = 0, 10, 15, 20) into the bilayer MX nanosheets. After the system is equilibrated, the acetone droplet containing 500 molecules is placed above the upper nanosheet to observe the behavior of acetone inserted into the bilayer MX nanochannel (Fig. S15†). The *x*–*y* size of the 2D nanochannel was set as 28.73 × 3.04 nm^2^. For both wetting and 2D confined systems, a periodic boundary condition (PBC) was applied in *x*- and *y*-directions, whereas an open boundary was used in the *z*-direction.

The parameters of bond, angle, dihedral, van der Waals interactions, and electrostatic interactions of PIL and acetone are described by the all-atom optimized potential for liquid simulations (OPLS-AA) force field,^33^ which has been used successfully to get the structures and properties of PIL and acetone.^34^ To accurately describe the electrostatic interactions of PIL, all charges were calculated by fitting the electrostatic potentials from first principles calculations. The atomic charge of PIL is calculated based on the two-stage restraint electrostatic potential (RESP) method using the Gaussian 09 D.01 program package. The ionic geometry and atomic charge were obtained with the 6-311+G basis set for all elements in PIL. Ti_3_C_2_T_*X*_ modified by the hydroxyl group was used as the model of MX nanosheets, where the Lennard-Jones (LJ) parameters and atomic charges used for MX were set as suggested by Xu *et al.*^35^ The nonbonding interactions between different atoms in the system include both electrostatic and van der Waals terms. The former one, the long-range coulombic interaction, was computed by using the particle-particle-particle-mesh (PPPM) algorithm. The latter one is described by using the 12-6 LJ potential. The Lorentz–Berthelot mixing rules are used to model the parameters between different atomic species, which are truncated at 1.2 nm.

All molecular dynamics (MD) simulations in this work are performed using the large-scale atomic/molecular massively parallel simulator (LAMMPS).^36^ The timestep for integrating Newtonian equations of motion is 2.0 fs, which is confirmed to guarantee energy conservation. Throughout the MD simulations, all MX nanosheets were rigid to maintain the stable planar structure. In the MD simulations, polymer droplets and acetone were relaxed in the NVT ensemble at a temperature (*T*) of 300 K, controlled by the Nosé–Hoover thermostat. After 10 ns, the system was in equilibrium, and five independent MD simulations were run for an additional 1 ns to collect data with the statistical average for analysing structures.

### Density functional theory (DFT) calculations

All first-principles calculations were performed using the Vienna *Ab initio* Simulation Package (VASP)^37^ with the projector augmented wave (PAW) method and Perdew–Burke–Ernzerhof (PBE) functional.^38^ The Semiempirical Grimme parameter DFT-D3 correction was applied to describe the van der Waals interaction.^39^ The cut-off energy for the plane wave basis was set as 500 eV. The 2 × 2 × 1 *k*-point meshe in the Brillouin zone was used for structural optimization. The vacuum layer was more than 1.5 nm along the *z*-direction to avoid interactions between neighboring images. The convergence criteria for energy and force were 1 × 10^−5^ eV and 0.01 eV Å^−1^, respectively.

## Conclusions

We have developed ultrafast yet adjustable bilayer actuators with a MXene layer and a polymer-modified MXene layer through a facile and flexible fabrication process. The deformation rate in response to acetone vapor is significantly superior to previously reported MXene-based actuators. Small- and wide-angle X-ray scattering (SAXS and WAXS, respectively) indicated a gradient in the degree of polymer intercalation in the polymer-MX active layer (gradient intercalation-dominated), which may be responsible for the counterintuitive and fast bending behaviour. The actuation mechanism is further elucidated by DFT and MD simulations, which suggest that the different diffusion rates of the two layers (gradient acetone diffusion-dominated) also contribute to the outstanding actuation performance. Moreover, this preparation concept is applicable to other functional polycations. The difference in bending and unbending speed is related to the amount of polymer coating, the swelling capability of the polymer, and the binding energy between the polymer and MX flakes. This gradient-structure design concept will broaden the structural library of MXene-based films and offer a practical approach to achieving customised actuation performance.

## Data availability

The data supporting this article have been included as part of the ESI.†

## Author contributions

Andi Di: conceptualization, methodology, investigation, data analysis, writing – original draft, and writing – review & editing. Chenlu Wang: methodology, investigation, writing – original draft. Hongyan He: conceptualization, methodology, investigation and writing – review & editing. Yanlei Wang: conceptualization and methodology. Miao Zhang: conceptualization, methodology, investigation and writing – review & editing. Lennart Bergström: conceptualization, methodology, investigation and writing – review & editing. Jiayin Yuan: conceptualization, methodology, investigation and writing – review & editing. Wentao Deng: conceptualization and writing – review & editing. Pierre Stiernet: investigation and data analysis.

## Conflicts of interest

There are no conflicts to declare.

## Supplementary Material

SC-OLF-D4SC04935G-s001

SC-OLF-D4SC04935G-s002

SC-OLF-D4SC04935G-s003

SC-OLF-D4SC04935G-s004

SC-OLF-D4SC04935G-s005

SC-OLF-D4SC04935G-s006

SC-OLF-D4SC04935G-s007

SC-OLF-D4SC04935G-s008

SC-OLF-D4SC04935G-s009

SC-OLF-D4SC04935G-s010
