# Supplementary material for: MXene-based solvent-responsive actuators with a polymer-intercalated gradient structure
Source: Chem Sci. 2024 Dec 3;16(5):2191–201. doi: 10.1039/d4sc04935g (PMC11629778; doi:10.1039/d4sc04935g)
Supplement: SC-016-D4SC04935G-s002 [file SC-016-D4SC04935G-s002.pdf]

Supplementary Information (SI) for Chemical Science.
This journal is © The Royal Society of Chemistry 2024

Supporting Information for

MXene-based Solvent-responsive Actuators with a Polymer-intercalated Gradient Structure

Andi Di,†[a] Chenlu Wang,†[b] Yanlei Wang,[c] Hongyan He,*[c] Wentao Deng,[d] Pierre Stiernet[a], Lennart Bergström*[a], Jiayin Yuan*[a] and Miao Zhang *[a]

a. Department of Materials and Environmental Chemistry, Stockholm University Stockholm, 114 18, Sweden
b. Beijing Key Laboratory of Ionic Liquids Clean Process, State Key Laboratory of Multiphase Complex Systems, CAS Key Laboratory of Green Process and Engineering, Institute of Process Engineering, Chinese Academy of Sciences, Beijing 100190, China
c. College of Chemistry and Chemical Engineering, Central South University, Changsha 410083, China

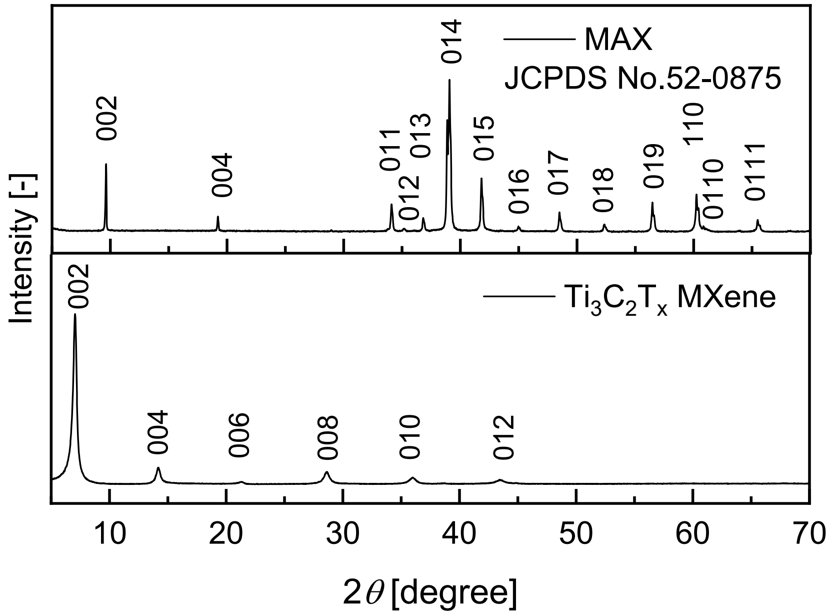

Figure. S1. XRD patterns of Ti_3AlC_2 (MAX) and the prepared $Ti_3C_2T_x$ MXene (MX).

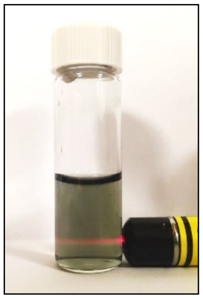

Figure S2. The Tyndall effect of MX aqueous dispersion.

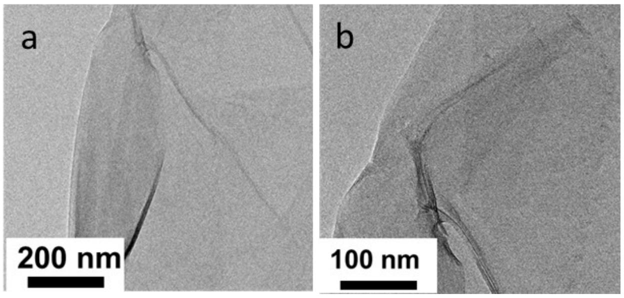

Figure S3. TEM images of (a) MX and (b) PDDA-MX flakes.

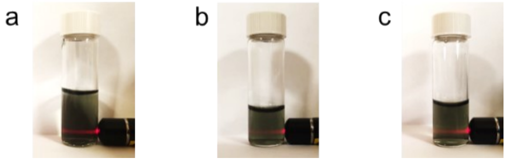

Figure S4. Tyndall effect of polymer-modified $Ti_3C_2T_X$ aqueous dispersions; (a) PIL-MX (b) PAH-MX and (c) PDDA-MX.

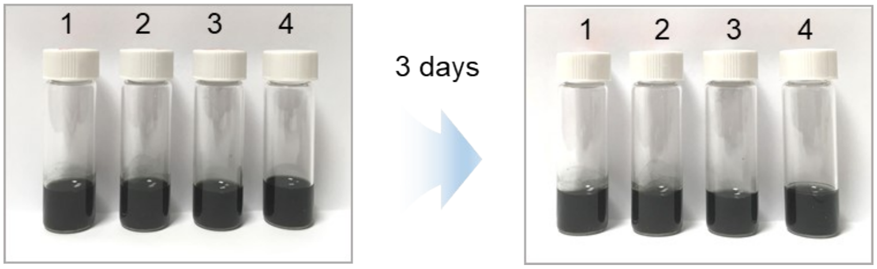

Figure S5. Photographs show the stability of colloidal dispersions of different samples (1) PIL-MX, (2) PAH-MX, (3) PDDA-MX, and (4) $Ti_3C_2T_X$ MXene in water.

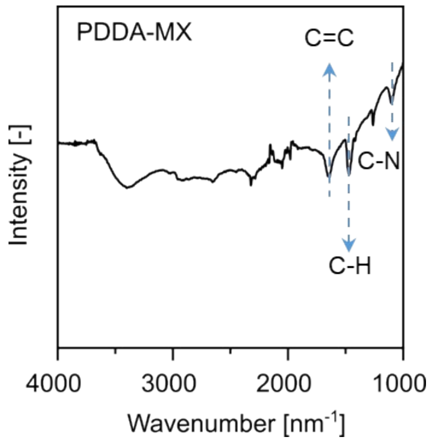

Figure S6. FTIR spectrum of PDDA-modified $Ti_3C_2T_X$ (PDDA-MX).

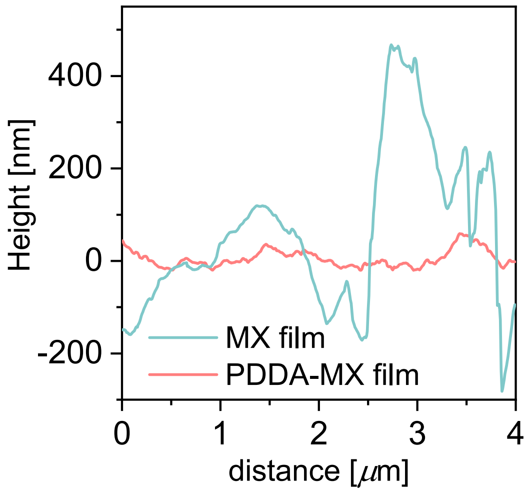

Figure S7. The height profiles of MX film and PDDA-MX film obtained from corresponding AFM images.

Table S1. Polarity comparison chart of the used organic solvents.

Solvent	Relative Polarity
Acetone	0.355
2-propanol	0.546
Diethyl Ether	0.117
Ethanol	0.654
Ethyl Acetate	0.228
Acetonitrile	0.460
Ethylene Glycol	0.790
Toluene	0.099
Hexane	0.009

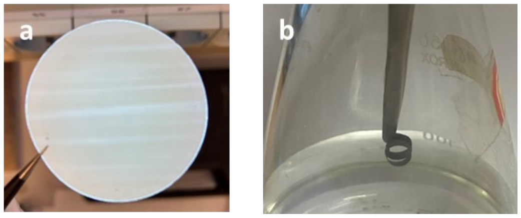

Figure S8. (a) A photograph of the used PVDF membrane after preparing the PDDA-MX/MX film, taken above a torch light to highlight its surface linear pattern. (b) The bending behavior of PDDA-MX/MX$_{(CNF)}$ film actuator prepared on a uniform CNF membrane in acetone vapor.

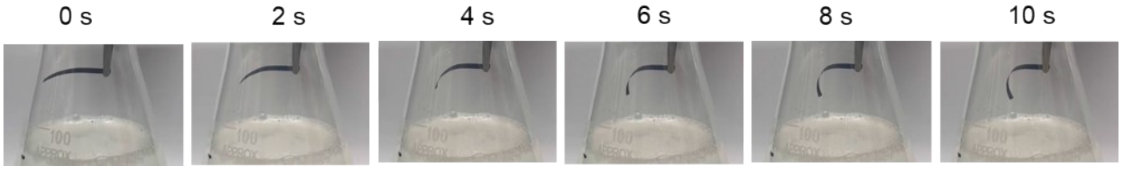

Figure S9. Deformation performance of PDDA-MX/MX film actuator at 97% relative humidity (saturated K_2SO_4).

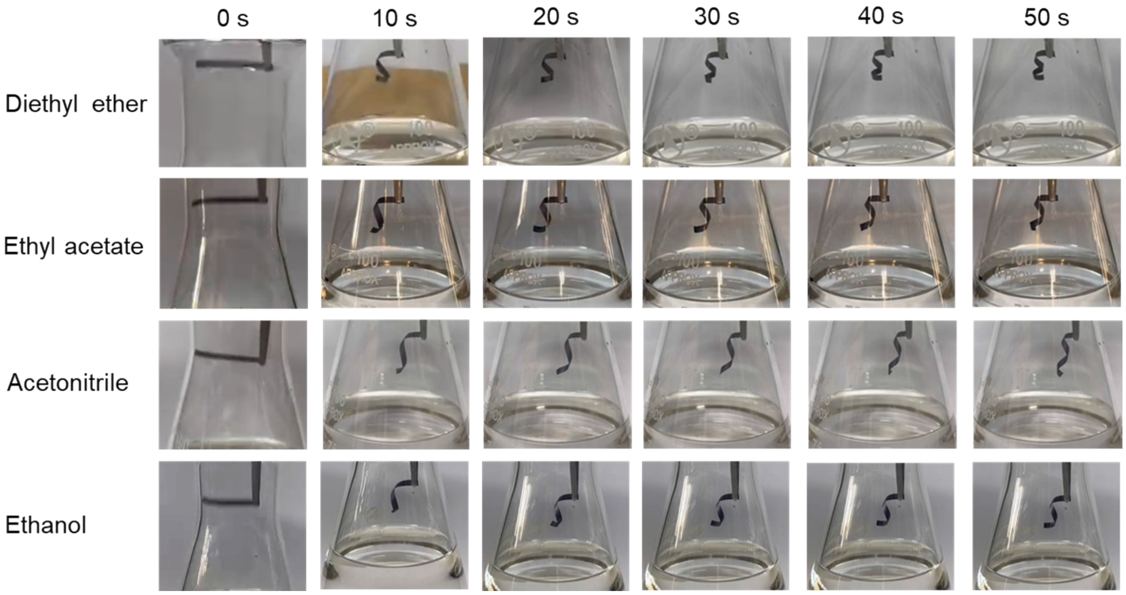

Figure S10. Time course profiles of the helical bending of the PDDA-MX/MX film in vapors of diethyl ether, ethyl acetate, acetonitrile, and ethanol.

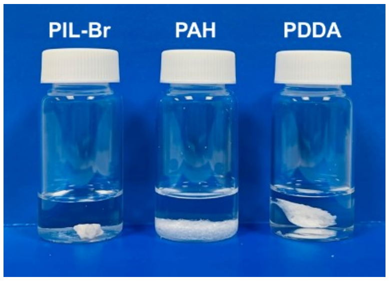

Figure S11. Solubility test of three kinds of polymers in acetone.

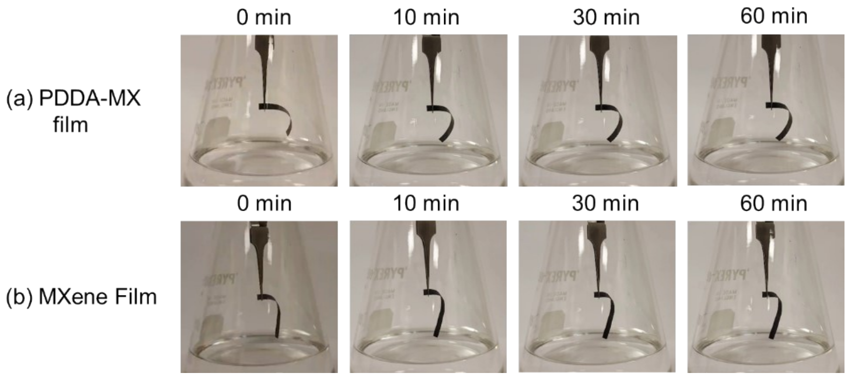

Figure S12. Time course profiles, (a) the PDDA-MX/MX film in acetone vapor, and (b) The MX film in acetone vapor.

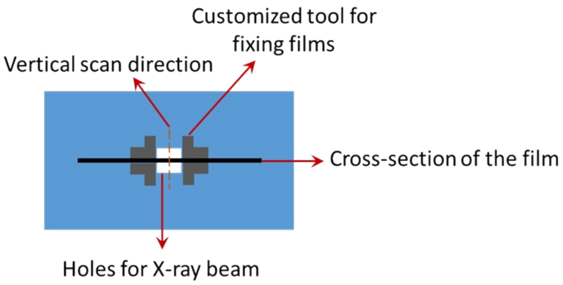

Figure. S13. The illustration of the set-up of the film sample on the sample holder.

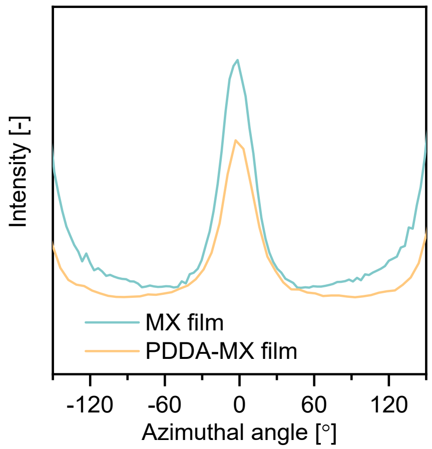

Figure S14. Comparison of the azimuthal plot of WAXS intensity of the (002) ring from MX film and PDDA-MX film, the Full Width at Half Maximum (FWHM) are 29.8 ° and 30.1 °, respectively.

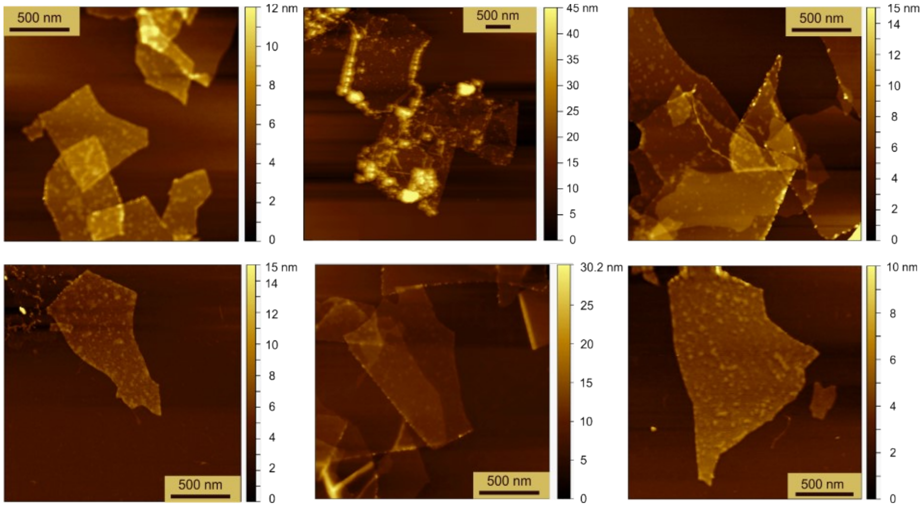

Figure S15. AFM image of PDDA-MX flakes.

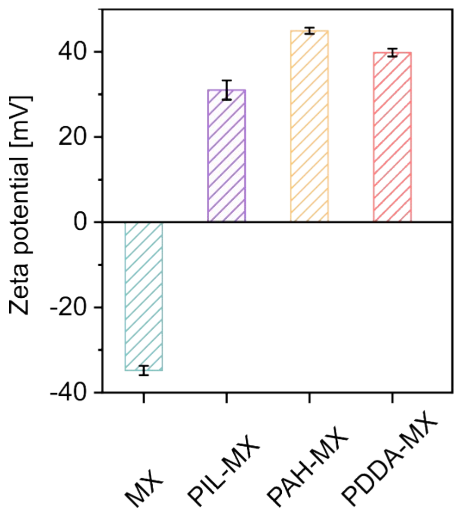

Figure S16. Zeta potentials of MXene (MX) and polymer-modified MXenes in water.

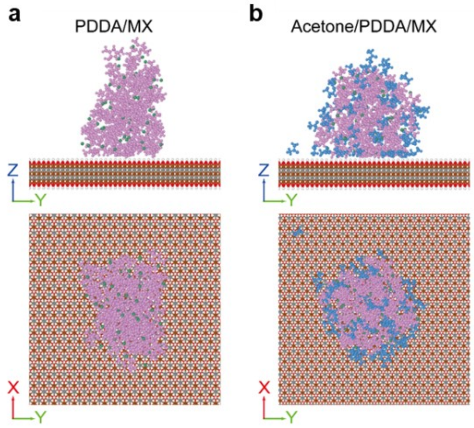

Figure S17. MD simulations. (a) The equilibrium configuration of the pure PDDA droplet on the MX surface, where pink and green colors represent polycations and anions of PDDA, respectively. (b) The equilibrium configuration of the acetone-doped PDDA droplet on the MX surface, where the blue color represents acetone.

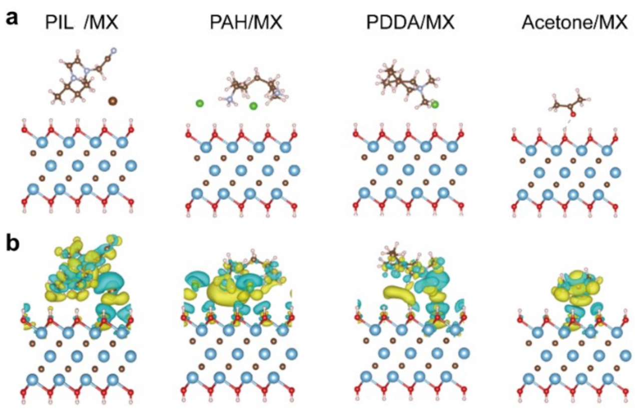

Figure S18. DFT calculations. (a) The configuration of PIL/MX, PAH/MX, PDDA/MX and acetone/MX for DFT calculations. (b) The charge density differences (CDD) of PIL/MX, PAH/MX, PDDA/MX and acetone/MX, where yellow and blue colors represent the accumulation and depletion of charge, respectively. The iso-surface level is 0.0007 e Å^{-3}.

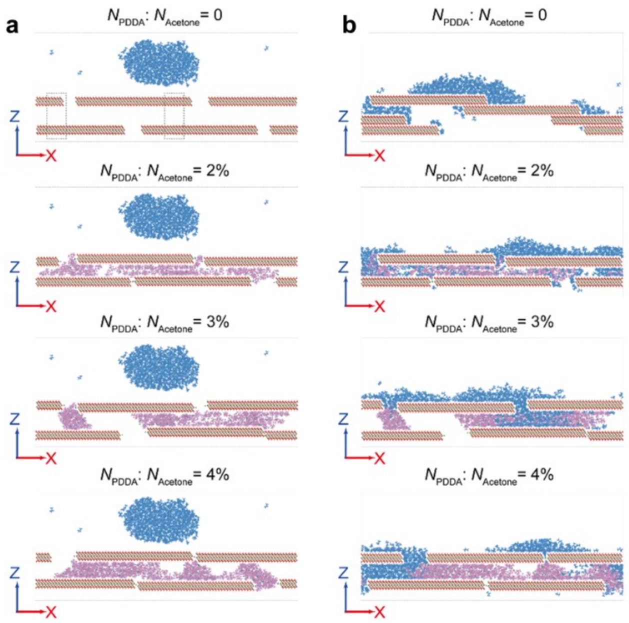

Figure S19. MD simulations. (A) Acetone molecules ($N_{Acetone}$ = 500) initially positioned above the pre-equilibrium PDDA-MX filled with different PDDA chains (N_{PDDA} = 0, 10, 15, and 20, corresponding to a N_{PDDA}:$N_{Acetone}$ ratio of 0, 2%, 3% and 4%, respectively). (B) The equilibrium configuration of acetone molecules inserted into the PDDA-MX.

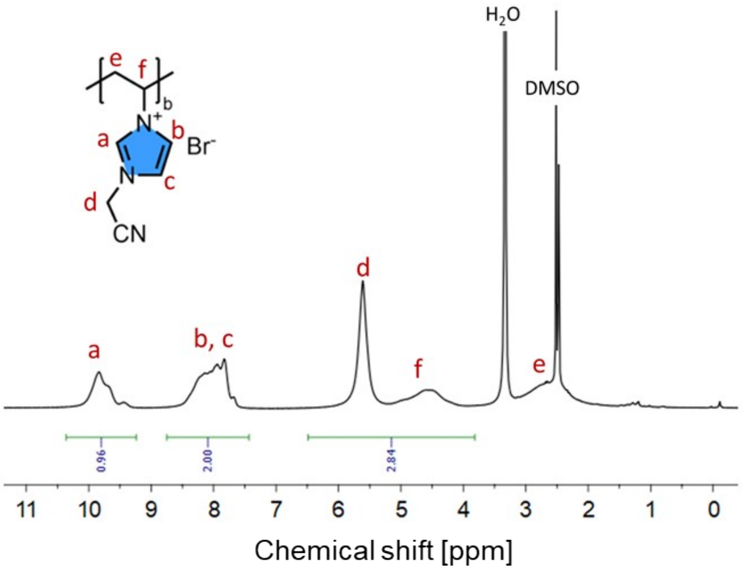

Figure S20. ^{1}H NMR spectrum. Poly(1-cyanomethyl-3-vinylimidazolium bromide) (PIL) in deuterated DMSO.

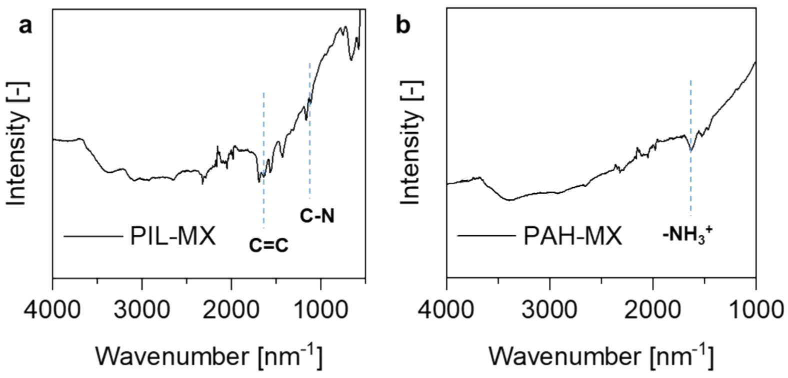

Figure S21. FTIR spectra. (A) PIL-modified MXene (PIL-MX) and (B) PAH-modified MXene (PAH-MX).

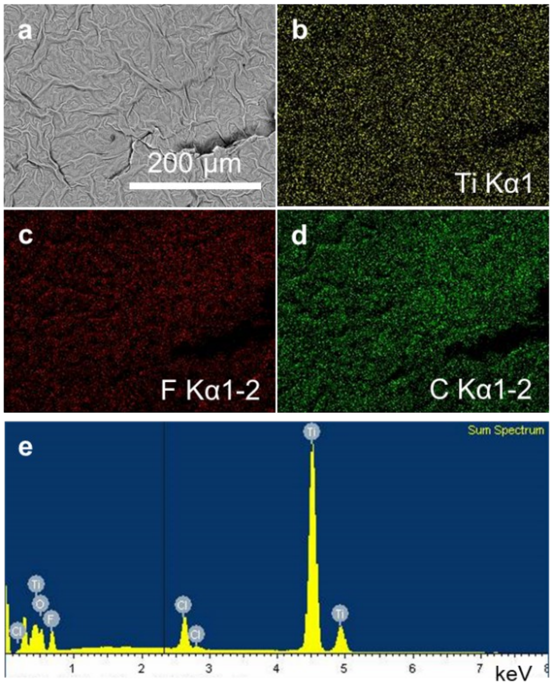

Figure S22. (a) SEM image, elemental mapping of (b) Ti, (c) F, (d) C, and (e) EDS spectrum of MXene film prepared through vacuum filtration.

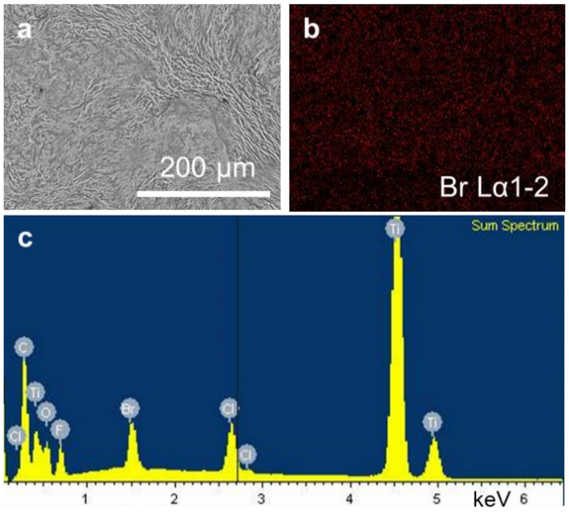

Figure S23. (a) SEM image, elemental mapping of (b) Br, and (c) EDS spectrum of PIL-MX film prepared through vacuum filtration.

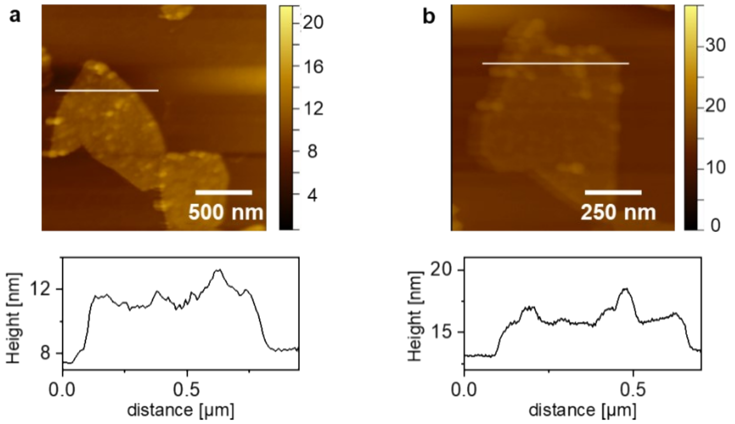

Figure S24. AFM images of (a) PIL-MX flakes and (b) PAH-MX flakes, and their corresponding height profiles.

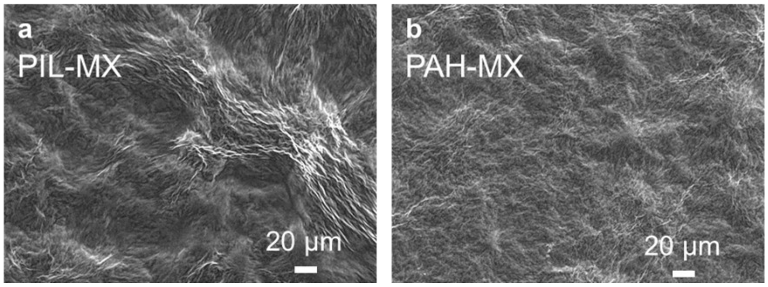

Figure S25. SEM images of the surface of (A) PIL-MX and (B) PAH-MX films.

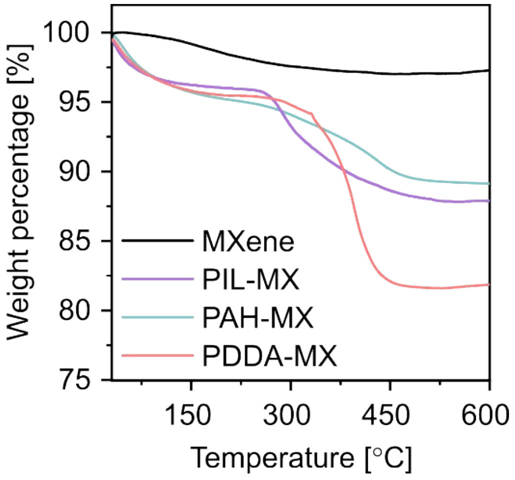

Figure S26. TGA curves of MXene and polymer-modified MXene (PIL-MX, PAH-MX and PDDA-MX) under Ar atmosphere.

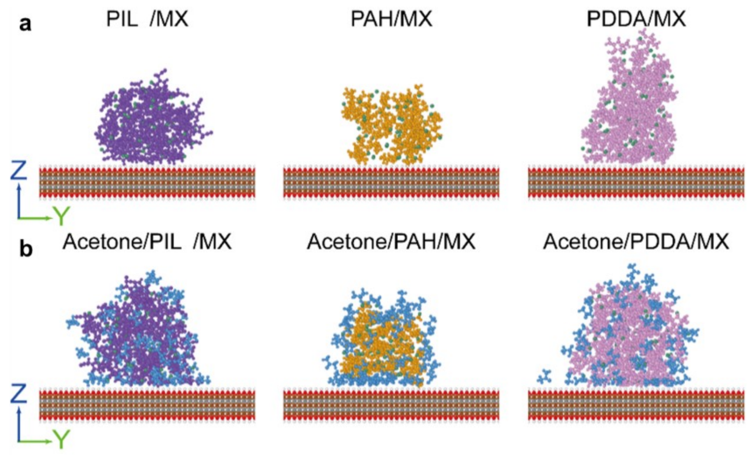

Figure S27. (a) The equilibrium configuration of three polymer droplets on the MX surface, where violet and orange colors represent polycations in PIL and PAH, respectively. (b) The equilibrium configuration of three acetone-doped polymer droplets on the MX surface.
